# Motivational Climate in Youth Football Players

**DOI:** 10.3390/bs8090083

**Published:** 2018-09-15

**Authors:** Manuel Castro-Sánchez, Félix Zurita-Ortega, José Luis Ubago-Jiménez, Irwin A. Ramírez-Granizo, Ramón Chacón-Cuberos

**Affiliations:** 1 Department of Education, University of Almería, 04120 Almería, Spain; mcastros@ual.es; 2 Department of Didactics of Musical, Plastic and Corporal Expression, University of Granada, 18071 Granada, Spain; felixzo@ugr.es (F.Z.-O.); irwin@correo.ugr.es (I.A.R.-G.); 3 Department of Integrated Didactics, University of Huelva, 21007 Huelva, Spain; ramon.chacon@ddi.uhu.es

**Keywords:** motivational climate, football, teenagers

## Abstract

(1) Background: In recent decades, the psychology of sport has gained special relevance in this field, due to the influence of psychological variables on sports performance and the regularity of sports practice. The aim of this research is to analyse the motivational climate of footballers. (2) Methods: This study uses a descriptive cross-sectional design on a sample of 156 adolescent football players, using an ad-hoc questionnaire for the recording of socio-demographic variables and the PMCSQ-2 questionnaire on motivational climate in sport. (3) Results: The results of the present investigation indicate that footballers are more oriented towards task than ego, sportsmen who compete in Honor Division being the those who are more oriented towards ego and those of National Division being more oriented towards task. (4) Conclusions: The main conclusion of this research is those who are the motivational climate is related to the division in which the players compete.

## 1. Introduction

Football is one of the most complex sports, in which technical, tactical, psychological, affective and social factors interact [1]. Football is considered a dynamic sport [2]. It is a sport with some characteristics that make it very attractive for both public and players. It is due to fact that it unites a series of simple movements that provoke emotions in the agents involved in this sport [3].

Motivation is one of the most analysed factors in sports psychology as it is one of the most influential factors in explaining human behaviour [4]. It is a crucial aspect in sport. Motivation directly influencing behaviour, changing its intensity, direction and attitude [5].

To address motivation in football, this research will focus on the motivational climate in sport, and a quick overview of Achievement Goals Theory [6,7], based on Deci and Ryan’s (1980) [8] Theory of Self-Determination, will be necessary.

It is commonly believed that the coach's main mission is to develop the technical and tactical skills of footballers. In the last two decades, psychological factors have gained special relevance in sports field. The latter having a close relationship with sports performance, as in the case of motivation [9]. Coaches are the ones who create motivational climates in their teams, they will promote orientations of their players towards task or towards ego [10,11].

One of the lines of research that has been researched in football has been the relationship between antisocial behaviour and the motivational climate [12]. We found studies that show the importance of environment created by coach on athlete behaviour when establishing antisocial environments.

In this regard, Miller, Roberts and Ommundsen (2005) [13] showed how a high ego-enlargement climate will encourage anti-social behaviour among footballers, while the task climate will promote pro-social behaviour that favours group cohesion and personal development [14,15], findings in the study of Cecchini, Méndez and Múñiz (2002) [16] about footballers show that task climate is related to greater intrinsic motivation.

The study by Torregrosa, Sousa, Viladrich, Villamarín and Cruz (2008) [17] maintains that motivational climate promoted by a football coach is directly related to the player’s perception of their performance on the pitch. Also determines the degree of commitment and fun of the players [18].

A study by García-Calvo, Leo, Martín and Sánchez (2008) [19] found that players with a task-oriented climate were more committed to playing sports. Likewise, Balaguer, Castillo and Duda (2003) [20] related involvement in the task to greater group cohesion, increasing the levels of commitment to the team and to sport. This aspect becomes vitally important when analyzing the higher categories, since in these categories, individual development seems to take precedence over group cohesion, so it is necessary for coaches to work on this cohesion through a task-oriented climate, as this will improve relations and understanding among the team's players [21].

The motivational climate perceived by footballers with anxiety levels has also been analyzed, finding in the study by García-Mas et al. (2011) [22] a directly proportional relationship between high anxiety levels and the ego-oriented climate.

Regarding sports performance, it is interesting to mention the study by Castillo, Balaguer and García-Merita (2007) [23] in which it was found that the football teams that obtained the highest scores in the task climate were in the top positions of the competition, while those that had the highest ego orientation levels were in the lowest positions [24].

This study aims to investigate the football players’ motivational climate in relation to various socio-demographic variables, considering these factors as conditioning factors of sporting activity to be analyzed. The motivational climate study is considered because it is an influential sports development factor. Usually affects performance, determining its effectiveness. This research aims to complement various studies carried out in the field of sport in other disciplines such as judo or sport itself.

## 2. Materials and Methods

### 2.1. Subjects and Design

The research was carried out in a descriptive cross-sectional study to analyse the possible dependence of the socio-demographic variables analysed on the motivational climate. The sample was composed of 178 young footballers (16–18 years old) belonging to the male sex and federated from Jaén, competing in the Spanish categories: Honor Division, National, Preferential, First Provincial and Second Provincial. For the sample selection, we use a convenience sampling was carried out according to the described divisions. Twenty-two questionnaires were eliminated as they were incomplete or poorly completed. Therefore, the final sample on which the research was carried out is made up of 156 football federated players between 16 and 18 years of age ($\bar{x}$= 16.96: SD = 0.773).

### 2.2. Measures

The instruments used in the study (included in the annexes) have been: 

Motivational Climate (PMCSQ-2). Extracted from the original version "Questionnaire Perceived Motivational Climate in Sport Questionnaire" by Newton, Duda and Yin (2000) [25] and adapted to Spanish and Physical Education by González-Cutre, Sicilia and Moreno (2008) [26], where through a Likert scale of five options ranging from 1 = Totally Disagree to 5 = Totally Agree, 33 items are assessed. This test is also established in two categories: Task Climate (TC) (cooperative learning, effort/improvement and important role) and Ego Climate (EC) (punishment for mistakes, unequal recognition and intra-team member rivalry).

This questionnaire has been chosen over others for its wide use in recent years in scientific research, considering it optimal for the study of motivational climate in sport, it has also been used in studies such as Castro (2016) [27] or Zurita, Zafra, Valdivia, Rodríguez, Castro and Muros (2017) [28].

Ad Hoc Questionnaire. We used our own questionnaire to record socio-demographic variables and those related to sports practice (studies, work, demarcation, division and years of federated practice).

### 2.3. Procedure

We contacted the team chairs to inform them about the nature of the study, explaining the objectives of the study and requesting their collaboration, and reporting on what the study was intended to do. Visits were arranged with the clubs to collect data, in which the researcher was always present in order to resolve doubts and ensure the correct completion of the questionnaires.

The data collection process lasted one and a half months, between March and April 2017. This research has respected the participants' right to confidentiality, following the 1975 Helsinki Declaration and Research Committee's ethical standards. In all cases, the informed consent of the parents or legal guardians was obtained, always respecting confidentiality.

### 2.4. Data Analysis

Statistical analysis of the data was performed using IBM SPSS software version 23.0. The distribution of the normalcy of the sample has been verified with the Kolmogorov-Smirnov and Saphiro-Wilk tests, and it was observed that the values followed a normal trend, so parametric tests were used. The basic descriptions were defined through frequencies and means. To see the differences between the variables, single-factor ANOVA was used, applying the Bonferroni test posteriori to check the intergroup differences.

## 3. Results

In the present study, which included a sample of 156 football players, it was determined that the age extract with the highest representation was 17 years old (40.4%; n = 63), followed by the subjects who were 16 years old (32.1%; n = 50), there were only 27.6% (n = 43) of 18-year-old participants.

Regarding the category in which the players played, the study had 26.3% (n = 41) of players competing in the Honor Division, followed by 22.4% (n = 35) in the First Provincial, 19.9% (n = 31) in the Second Provincial, followed by 17.9% (n = 28) in the National, and finally, 13.5% (n = 21) in the Preferred category.

According to the demarcation of the players surveyed, 36.5% (n = 57) were defenders, 25.0% (n = 39) midfielders, 21.2% (n = 33) played as forwards, and only 17.3% (n = 27) were goalkeepers.

When we analyse the years that the players in the sample have been football federated, we see that the majority (85.9%; n = 134) have been playing in a federated way for more than five years, followed by 9.0% (n = 14) for three to four years, and only 2.6% (n = 4) for one to two years, identical to the figures for players who have been federated for less than a year.

The majority of the footballers analysed (94.2%; n = 147) do not work, while only 5.8% (n = 9) are currently working.

About the studies level of sportsmen and women who make up the study, it has been found that almost half of the sample (47.4%; n = 74) have high school studies, followed by 32.1% (n = 50) who only have compulsory secondary education studies, 12.8% (n = 20) are in vocational training, and only 7.7% (n = 12) are in university studies.

When analysing the Motivational Climate of the players, higher scores were found in the TC (3.87 ± 0.53) and in the categories of cooperative learning (3.76 ± 0.72), Effort/Improvement (3.94 ± 0.56) and important role (3.88 ± 0.69). The scores are lower in the EC (2.97 ± 0.53) and its categories punishment for mistakes (3.00 ± 0.69), unequal recognition (2.75 ± 0.80) and intra-team member rivalry (3.34 ± 0.56).

All the socio-demographic and motivational climate characteristics of the sample mentioned above are summarized in Table 1.

When analyzing the Motivational Climate and the category in which the athletes compete (Table 2), statistical differences have been found (*p* ≤ 0.05) in all variables studied, except in punishment for mistakes (*p* = 0.071).

Statistical differences were observed between five football categories (*p* < 0.05). When the comparisons were made by pairs, it was observed that Honor and Preferred Divisions players presented higher values for EC than the Second Provincial players (3.11 ± 0.51 Honor Division, 3.19 ± 0.51 Preferred, 2.72 ± 0.49 Second Provincial; *p* < 0.05). For the Unequal Recognition category, significant differences between groups were also observed (*p* < 0.05). The peer comparisons showed that Second Provincial (2.40 ± 0.72) had lower values than Preferred Provincial (3.23 ± 0.79) and First Provincial (2.96 ± 0.89). Preferred division presented higher values than National (2.47 ± 0.68). Regarding the category intra-team member rivalry, statistical relationships between groups have also been found (*p* < 0.05). When making the comparisons by pairs, it was found that Second Provincial (3.00 ± 0.58) presented values lower than Honor Division (3.50 ± 0.50), National Division (3.41±.48) and Preferential Division (3.47 ± 0.46).

TC statistical relations have also been found (*p* ˂ 0.05) when making the comparisons by pairs, It was found that National (4.13 ± 0.41) obtains higher values than Second Provincial (3.66 ± 0.48). When analyzing the relationships with the Important Role category, there are also statistically significant relationships (*p* = 0.016), proving after comparison by pairs that National category (4.16 ± 0.481) have higher scores than Second Provincial (3.56 ± 0.70).

As can be seen in Table 3, which analyzes the differences between the Motivational Climate and its categories with the study level of athletes, no statistically significant differences have been found (*p* ≥ 0.05).

When analyzing the differences between the Motivational Climate and players years have been federated no statistical association (*p ≥* 0.05) has been found between any category.

Finally, when relating the Motivational Climate and its categories according to the athlete’s demarcation, no statistically significant differences have been found (*p ≥* 0.05).

Statistical differences were only found (*p* ≤ 0.05) in the category Intra-team member rivalry in terms of the differences between the Motivational Climate and whether or not footballers work (Table 4), with the figures being higher (3.36 ± 0.54) for working sportsmen and men who do not (2.96 ± 0.78). No statistically significant differences have been found in other categories of Motivational Climate (*p ≥* 0.05) when compared to whether or not athletes work.

## 4. Discussion

This research was carried out with a total of 156 players between the ages of 16 and 18. The players competed in Honor Division, National, Preferential, First Provincial and Second Provincial categories. The studio has goalkeepers, defenders, midfielders and forwards. Most of the footballers analysed had been playing this sport in a federated way for more than five years and did not work, having a professional training and high school education level.

The athletes analyzed are more oriented towards task than ego, the athletes who compete in the Honor Division being those who are more oriented towards ego and those of Nacional those who are more oriented towards task. These data are consistent with all the studies consulted [12,24,29,30,31], both in football and in other sports contexts. This is explained by the highest competitive levels, sportsmen and women focus on ego with the purpose of demonstrating their abilities and overcoming their opponents in order to win in competitions. While players at lower levels focus more on progress and personal improvement, considering competitions as learning situations [27].

Although there is no statistical association, when the Motivational Climate is related to athlete’s study level, there is a trend that shows a direct relationship between ego orientation and lower levels of studies. While the players with higher academic training are oriented towards task, except in the case of university students, who find the lowest figures in this type of orientation. This can be explained because with less academic training, the players acquire fewer social skills related to effort and self-improvement. While those who continue their studies understand the importance of effort and perseverance.

These data differ from those found in the study of Sánchez-Alcaráz and Gómez-Mármol (2014) [32] and Sánchez-Alcaráz, Gómez-Marmol and Más (2016) [33], who obtained data in their studies that indicate a proportional increase in the EC levels when increasing their studies level. This suggests that this is due to a greater sense of age-related motor competence [34].

No statistical association is found when analyzing the relationship between the motivational climate and the years that footballers have been federated. Although there is a tendency to increase their orientation towards ego in sportsmen and women who have been federated for more years. It may be due to the fact that players who have been practicing this sport for longer try to demonstrate their abilities in order to get into higher categories and acquire the status of professional sportsman. These data are consistent with most of consulted investigations [35,36,37].

The players who work have a greater sense of rivalry with their teammates than those who do not work. No studies have been found that analyze the relationship between working or not working and the motivational climate, so the results cannot be compared. This occurs because the footballer is inserted into the work market moves in a more competitive context, being aware that group although work improves the final result. There is rivalry between the members of the group to highlight and get the best positions, both in the work and sports context [38,39].

Goalkeepers, midfielders and forwards are more task oriented than ego oriented. It is reversed in the case of defenders. This relationship is not significant, although there is a trend. No similar studies have been found with which to compare the data. Although it is felt that this tendency found may be due to the fact that the situations faced by the defenders are usually those of opposition and greater contact. It leads to attitudes oriented towards skill demonstration. The strikers might be thought to be the most ego-oriented of all the players, though.

This study provides important data on anxiety levels and the Motivational Climate of adolescent football players. It serves as a basis for further studies and an intervention program that reduce the anxiety levels found in athletes and improves the Motivational Climate. The program would serve as an athlete’s orientation more towards task than ego, as these are lower categories in which footballers are still being trained as athletes.

Although the research does not allow us to establish recommendations for the intervention programs in an exhaustive manner. This is a descriptive cross-sectional study with a scarce sample. It is necessary to pay attention to Motivational Climate promoted by coaches at lower football levels, which should promote task-oriented climates because, even though athletes are in competitive categories. They are still at development and improvement ages, so that an ego-oriented climate would harm their evolution as athletes. This can be done through intervention programs designed to reorient the Motivational Climate in that coaches promote use of communication tools designed for this purpose that coach can use to create a task-oriented climate. He can use changes in the discourse towards the athletes, always providing positive feedback, rewarding effort and personal improvement before the result and avoiding comparisons between the team's athletes.

## 5. Conclusions

As a result of the present investigation, it has been determined that most of them had been playing football for more than five years in a federated way and did not work, and with most of them having a High School education level.

The players are being more oriented towards task than ego, the sportsmen who compete in Honor Division being those who are more oriented towards ego and those of National being those who are more oriented towards task. The players who work tested have a greater sense of rivalry with their teammates than those who do not work.

## Figures and Tables

**Table 1:** Descriptive analysis.

**Age**	16	32.1% (n = 50)	
	17	40.4% (n = 63)	
	18	27.6% (n = 43)	
**Category**	Honor Division	26.3% (n = 41)	
	National	17.9% (n = 28)	
	Preferred	13.5% (n = 21)	
	First provincial	22.4% (n = 35)	
	Second provincial	19.9% (n = 31)	
**Demarcation**	Goalkeeper	17.3% (n = 27)	
	Defender	36.5% (n = 57)	
	Midfielder	25.0% (n = 39)	
	Forward	21.2% (n = 33)	
**Years federated**	First year	2.6% (n = 4)	
	Between 1 and 2 years	2.6% (n = 4)	
	Between 3 and 4 years	9.0% (n = 14)	
	Over 5 years	85.9% (n = 134)	
**Work**	No	94.2% (n = 147)	
	Yes	5.8% (n = 9)	
**Study level**	ESO	32.1% (n = 50)	
	Baccalaureate	47.4% (n = 74)	
	Professional Training	12.8% (n = 20)	
	University	7.7% (n = 12)	
		$\bar{x}$	σ
**Motivational Climate**	**Ego Climate**	2.97	0.53
	Punishment for mistakes	3.00	0.69
	Unequal recognition	2.75	0.80
	Intra-team member rivalry	3.34	0.56
	**Task Climate**	3.87	0.53
	Cooperative learning	3.76	0.72
	Effort/improvement	3.94	0.56
	Important role	3.88	0.69

**Table 2:** Motivational Climate and football division.

	Football Division										*F*	Sig.
	Honor Division (n = 41)		National (n = 28)		Preferred (n = 21)		First Provincial (n = 35)		Second Provincial (n = 31)			
	$\bar{x}$	σ	$\bar{x}$	σ	$\bar{x}$	σ	$\bar{x}$	σ	$\bar{x}$	σ		
Ego Climate	3.11*	0.51	2.80	0.45	3.19 *	0.51	3.01	0.57	2.72	0.49	4.41	0.002
Punishment for mistakes	3.26	0.77	2.82	0.51	2.97	0.54	2.90	0.81	2.94	0.58	2.20	0.071
Unequal recognition	2.80	0.72	2.47 #	0.67	3.23 *	0.78	2.96 *	0.88	2.40	0.71	5.63	0.000
Intra-team member rivalry	3.50*	0.50	3.41 *	0.48	3.47 *	0.46	3.31	0.62	3.00	0.58	4.48	0.002
Task Climate	3.98	0.50	4.13 *	0.41	3.82	0.61	3.77	0.57	3.66	0.48	3.95	0.004
Cooperative Learning	3.92	0.65	4.01	0.62	3.61	0.79	3.67	0.75	3.53	0.72	2.61	0.035
Effort/Improvement	4.03	0.54	4.18	0.46	3.89	0.58	3.80	0.60	3.78	0.55	2.85	0.026
Important role	3.95	0.68	4.16*	0.48	3.91	0.84	3.83	0.65	3.56	0.70	3.16	0.016

* σ = Standard Deviation. * = Second provincial differences. #= Preferred differences.

**Table 3:** Motivational Climate, studies, Federated years and position.

	Studies									*F*	Sig.
	ESO (n = 50)		Baccalaureate (n = 74)			Professional Training (n = 20)		University (n = 12)			
	$\bar{x}$	σ	$\bar{x}$	σ	$\bar{x}$	σ	$\bar{x}$		σ		
**Ego Climate**	3.01	0.55	2.98		0.53	2.88	0.47	2.85	0.56	0.49	0.686
**Punishment for mistakes**	3.13	0.69	2.95		0.64	2.89	0.76	2.93	0.81	0.95	0.415
**Unequal recognition**	2.75	0.91	2.81		0.79	2.68	0.66	2.58	0.59	0.35	0.784
**Intra-team member rivalry**	3.34	0.57	3.36		0.55	3.26	0.64	3.27	0.54	0.22	0.879
**Task Climate**	3.85	0.54	3.90		0.53	3.96	0.45	3.65	0.62	0.94	0.420
**Cooperative Learning**	3.82	0.74	3.71		0.73	3.90	0.69	3.60	0.58	0.65	0.581
**Effort/Improvement**	3.88	0.57	3.99		0.56	4.03	0.46	3.71	0.65	1.17	0.322
**Important role**	3.84	0.68	3.95		0.67	3.88	0.71	3.60	0.80	1.01	0.390
	**Federated Years**									***F***	**Sig.**
	**First Year** **(n = 4)**		**1–2 Years** **(n = 4)**		**3–4 Years** **(n = 14)**		**Over 5 Years** **(n = 134)**				
	$\bar{x}$	**σ**	$\bar{x}$		**σ**	$\bar{x}$	**σ**	$\bar{x}$	**σ**		
**Ego Climate**	2.67	0.41	2.76		0.55	2.90	0.37	2.99	0.55	0.72	0.539
**Punishment for mistakes**	2.85	0.44	2.80		0.43	2.91	0.65	3.02	0.70	0.28	0.834
**Unequal recognition**	2.37	1.14	2.45		1.14	2.77	0.44	2.77	0.81	0.51	0.674
**Intra-team member rivalry**	3.00	0.47	3.33		1.18	3.16	0.59	3.37	0.54	1.05	0.371
**Task Climate**	3.88	0.36	3.91		0.43	3.71	0.44	3.89	0.55	0.45	0.714
**Cooperative Learning**	3.68	0.51	3.68		0.87	3.69	0.56	3.77	0.74	0.08	0.968
**Effort/Improvement**	4.00	0.38	3.85		0.20	3.72	0.51	3.96	0.58	0.79	0.497
**Important role**	3.87	0.66	4.25		0.61	3.73	0.60	3.88	0.70	0.59	0.620
	**Position**									***F***	**Sig.**
	**Goalkeeper (n = 27)**		**Defender (n = 57)**		**Midfielder (n = 39)**		**Forward (n = 33)**				
	$\bar{x}$	**σ**	$\bar{x}$	**σ**	$\bar{x}$	**σ**	$\bar{x}$	**σ**			
**Ego Climate**	2.87	0.53	3.02		0.53	2.98	0.61	2.93	0.43	0.55	0.649
**Punishment for mistakes**	2.91	0.68	3.02		0.58	3.00	0.80	3.04	0.73	0.20	0.893
**Unequal recognition**	2.60	0.78	2.80		0.83	2.86	0.83	2.67	0.72	0.76	0.513
**Intra-team member rivalry**	3.34	0.45	3.46		0.52	3.20	0.58	3.29	0.66	1.72	0.164
**Task Climate**	3.90	0.52	3.86		0.59	3.86	0.47	3.89	0.53	0.06	0.979
**Cooperative Learning**	3.63	0.69	3.82		0.71	3.70	0.74	3.84	0.73	0.61	0.604
**Effort/Improvement**	3.93	0.63	3.91		0.64	3.97	0.46	3.95	0.50	0.08	0.966
**Important role**	4.12	0.45	3.82		0.80	3.82	0.63	3.85	0.70	1.38	0.250

**Table 4:** Motivational Climate according to work.

	Work				*F*	Sig.
	No (n = 147)		Yes (n = 9)			
	$\bar{x}$	σ	$\bar{x}$	σ		
Ego Climate	2.72	0.63	2.98	0.52	2.08	0.151
Punishment for mistakes	2.77	0.77	3.01	0.68	1.00	0.317
Unequal recognition	2.55	0.98	2.77	0.79	0.61	0.435
Intra-team member rivalry	2.96	0.78	3.36	0.54	4.38	0.038
Task Climate	3.61	0.89	3.89	0.50	2.33	0.128
Cooperative Learning	3.63	0.99	3.77	0.70	0.29	0.588
Effort/Improvement	3.85	1.03	3.94	0.53	0.20	0.648
Important role	3.16	0.93	3.92	0.65	10.8	0.001

## Limitations and Future Research Lines

As main limitations we can emphasize that the ideal would have been to make a longitudinal study. In addition, the sample could have been extended to the national context to make it representative. Likewise, as future research lines, we want to extend the study throughout the Spanish context.
